# Mss2 shapes the virulence of *Candida albicans* through reactive oxygen species (ROS) and calcium signaling, independent of direct transcriptional control

**DOI:** 10.1080/21505594.2025.2590329

**Published:** 2025-11-20

**Authors:** Cai-Ling Ke, Hsiao-Yen Tsai, Ajinkya Kulkarni, Richard J. Bennett, Ching-Hsuan Lin

**Affiliations:** a Department of Biochemical Science and Technology, College of Life Science, National Taiwan University, Taipei, Taiwan; b Department of Molecular Microbiology and Immunology, Brown University, Providence, RI, USA

**Keywords:** *Candida albicans*, *MSS2*, mitochondria, calcium–ROS crosstalk, invasive growth, fungal virulence

## Abstract

Mitochondrial function is essential for virulence in *Candida albicans*, yet the mechanism by which mitochondria influence pathogenesis remains largely undefined. Here, we reveal that the mitochondrial-associated factor Mss2 controls invasive growth through the regulation of calcium–reactive oxygen species (ROS) homeostasis. Deletion of *MSS2* results in impaired invasive growth on solid media without affecting hyphal formation in liquid media, indicating that Mss2 controls contact-specific responses. We demonstrate that the regulation of these processes by Mss2 is linked to the regulation of cytosolic calcium levels and cellular ROS production. Furthermore, transcriptomic profiling identified *MSS2*-regulated genes, including *UME6*, *SAC1*, *RIM8*, and *ORF19.1841*, whose expression is dependent on calcium and ROS levels. Restoration of invasive phenotypes through exogenous ROS confirms the functional significance of this calcium-ROS circuit. In systemic infection models, similar to *mss2Δ*, the Mss2 downstream genes exhibit severe virulence defects. Together, this work is the first to show that mitochondrial regulation of a coordinated calcium-ROS circuit is required for invasive hyphal growth and virulence in *C. albicans*. These findings refine our understanding of fungal invasion and virulence and reveal that targeting mitochondrial signaling could be an important area for antifungal therapeutic interventions.

## Introduction

*Candida albicans* is an opportunistic fungal pathogen that typically inhabits the human body as a commensal organism [1–3]. However, it has the potential to cause severe infections, especially in immunocompromised individuals, such as those with HIV/AIDS or organ transplant recipients [1–3]. The ability of *C. albicans* to colonize various niches in the human body and to cause life-threatening diseases has been closely linked to its phenotypic plasticity [4–6]. This species can rapidly react to a wide range of environmental stimuli and change its morphology to promote forms more suited to colonization or to disease. For example, yeast cells are optimal for circulating in the bloodstream, whereas hyphal cells are better suited for tissue invasion through the secretion of proteases and the peptide toxin candidalysin [5–7].

The yeast-to-hypha transition is a well-established virulence factor in *C. albicans*, and its complex regulation is summarized in several review articles [5,6,8,9]. *C. albicans* cells typically exist in the single-celled yeast form but transition to the multicellular hyphal form in response to key host cues, including elevated temperature, CO_2_ and N-acetyl glucosamine [5,6,8,9]. However, the ability to form invasive filaments, rather than merely hyphal formation in liquid environments, is potentially a more critical determinant of *C. albicans* pathogenicity. In line with this, we previously showed that deletion of *MSS2* does not affect hyphal formation in liquid culture but severely impairs invasive growth into agar plates and virulence in a systemic model [10]. Notably, a number of genes can impact hyphal development both upon surface contact and under liquid culture conditions, including *MKC1*, *BUD2*, *RAS1*, *CDC24*, and *RSR1* [11–13]. In addition, several factors impact invasive growth without altering hyphal morphology in suspension, such as *RHR2*, encoding a glycerol 3-phosphatase, which is essential for maintaining intracellular turgor pressure for adhesion and biofilm formation yet is dispensable for hyphal development [14,15]. Likewise, the cell wall-associated protein Dfi1 governs invasive growth and virulence via activation of the transcription factors Sef1 and Czf1 and is essential for surface invasion but not for hyphal formation under liquid culture conditions [16,17]. Bcr1, a transcription factor, is similarly required for adhesion, biofilm development, and virulence, yet *bcr1Δ* null mutants retain normal hyphal growth in liquid media [18,19]. Collectively, these findings highlight that contact-dependent filamentation and associated invasive programs are distinct from general hyphal morphogenesis and yet crucial for tissue invasion and pathogenicity in *C. albicans*.

Calcium homeostasis has been closely linked to contact-dependent hyphal growth in *C. albicans* [16,17]. Upon surface contact, increased calcium levels induce calmodulin binding to Dfi1p and subsequently activate Cek1p to initiate invasive hyphal development [17]. Moreover, the Cch1-Mid1 channel complex in the calcineurin signaling pathway is also involved in calcium maintenance, as deletion of *CCH1* or *MID1* displayed invasive growth and virulence defects [20]. These studies implicate calcium signaling as playing a critical role in invasive growth and pathogenicity in *C. albicans*.

Mitochondria also play a key role in calcium homeostasis [21,22]. These organelles support cellular energy metabolism by generating ATP and regulating vital physiological processes, including programmed cell death, calcium homeostasis, and redox balance [23–26]. In fact, variations in mitochondrial calcium levels cause transient membrane depolarization, resulting in hyperpolarization and enhancing ATP generation and increasing ROS output [25,27,28]. Mitochondrial calcium levels can therefore serve as a vital link with ROS dynamics. Although the interaction between ROS and calcium within mitochondria has been widely recognized as a contributor to cellular damage under pathological conditions in mammalian cells, increasing evidence points to their cooperative role in maintaining cellular homeostasis [29–31]. Mitochondrial function is crucial for invasive growth in *C. albicans*, as ATP is needed for hyphal formation and for enzyme secretion to degrade host tissues [24,32]. However, the interplay between mitochondria, calcium, and ROS signaling in *C. albicans* has not been addressed, including how mitochondrial dysfunction affects contact-dependent invasive hyphal growth.

Our previous study showed that the loss of a mitochondrial-related gene, *MSS2*, results in normal hyphal formation in liquid media but profound defects in both invasive potential and virulence [10]. In this study, we reveal that *MSS2* regulates the expression of *UME6*, *RIM8*, *SAC1*, and *ORF19.1841* genes through its effects on calcium-ROS homeostasis. These downstream genes then play critical roles in mediating invasive growth and virulence in *C. albicans*. More importantly, this study is the first to reveal that *C. albicans* contact-dependent invasive behavior requires coordinated regulation of mitochondrial function, calcium signaling, and ROS levels, providing new insights into the complex interplay that drives fungal pathogenesis.

## Materials and methods

### Media and reagents

The media used in this study included YPD medium (2% Bacto peptone, 1% yeast extract, and 2% dextrose), YPD supplemented with 200 μg/mL nourseothricin (Werner BioAgents, Jena, Germany), and Roswell Park Memorial Institute 1640 (RPMI 1640) medium supplemented with 0.165 M MOPS and 2% glucose [33]. RPMI 1640 supplemented with 1 mM H_2_O_2_, 5 mM H_2_O_2_, 15 mM H_2_O_2_, 200 μM glutathione, or 0.6 mM EGTA for spotting and flow cytometry analyses.

### Plasmid and strain constructions

The *C. albicans* strains and oligonucleotides used in this study are listed in Table S1 and Table S2, respectively. For the construction of gene knockout strains, the 5′ and 3′ DNA flanking regions of the *UME6*, *SAC1*, *RIM8*, *ORF19.1841* and *DFI1* were PCR-amplified using primers 2255/2256–2257/2258, 2351/2352–2353/2354, 2335/2336–2337/2338, 2295/2296–2297/2298 and 2070/2071–2074/2075, respectively. The 5′ and 3′ PCR products *UME6*, *SAC1*, *RIM8* and *ORF19.1841* were digested with *Apa*I/*Xho*I and *Sac*II/*Sac*I, respectively, whereas the 5′ and 3′ PCR products of *DFI1* were digested with *Kpn*I/*Apa*I and *Sac*II/*Sac*I, and subsequently cloned into the plasmid pSFS2A [34] to generate the gene disruption constructs pSFS-UME6 KO, pSFS-SAC1 KO, pSFS-RIM8 KO, pSFS-ORF19.1841 KO and pSFS-DFI1 KO, respectively. The constructs for knockout *UME6*, *SAC1*, *RIM8* and *ORF19.1841* were linearized using *Apa*I/*Sac*I, and the plasmid for knockout *DFI1* was digested with *Kpn*I/*Sac*I. Each sample was then transformed into the wild-type *C. albicans* strain SC5314 to generate heterozygous deletion strains. The *SAT1* gene was recycled by growth on YP supplemented with 2% maltose. The marker-free heterozygous strains were then subjected to a second round of transformation with the same deletion construct to obtain homozygous knockout strains.

To generate complementation strains, the endogenous promoter and open reading frame (ORF) fragments of *UME6*, *SAC1*, *RIM8* and *ORF19.1841* were PCR-amplified using primers 2259/2260, 2355/2356, 2339/2340 and 2299/2300, respectively. The PCR products were digested with *Apa*I/*Xho*I and inserted into the plasmid pSFS2A to produce pSFS-UME6 AB, pSFS- SAC1 AB, pSFS-RIM8 AB, and pSFS-ORF19.1841 AB add-back plasmids. Each plasmid was then linearized with a gene-specific restriction enzyme, *Bsm*I for *MSS2*, *Sma*I for *UME6*, *Pml*I for *SAC1*, *Bcl*I for *RIM8*, and *Xba*I for *ORF19.1841* and then introduced into the corresponding mutant strains.

To construct the Mss2-Neon-NAT^R^ *C. albicans* strain (Mss2-mNeonGreen), SC5314 was transformed with a pMSS2-NEON-NAT^R^ plasmid. The *MSS2* open reading frame (ORF), the NEON sequence, and the downstream sequence of *MSS2* were amplified using primer pairs 9130/9131, 9133/9134, and 9132/9135, respectively. The PCR products were then cloned into the pGGA-Select backbone by *Bsa*I-HF V2 restriction enzyme digestion and simultaneous ligation with a ligase enzyme to generate the pMSS2-NEON-NAT^R^ plasmid. The transformant was finally digested with the restriction enzyme *Pac*I and transformed into SC5314 to generate the Mss2 labeling strain.

To construct gene overexpression strains, the open reading frames (ORFs) of *SAC1*, *ORF19.1841*, *UME6*, and *RIM8* were PCR-amplified using primer pairs Oligo 2664/2665, 2668/2669, 2666/2667, and 2670/2671, respectively. The PCR products were digested with the restriction enzymes (*Sal*I/*BamH*I for *SAC1*, *Not*I/*Stu*I for *ORF19.1841* and *UME6*, and *Not*1/*AflI*I for *RIM8*) and subsequently ligated into the pNIM1 plasmid [35] to generate pNIM-*SAC1* OE, pNIM-*ORF19.1841* OE, pNIM-*UME6* OE, and pNIM-*RIM8* OE constructs. Each plasmid was then linearized using *SacI*I and *PspOM*I restriction enzymes and introduced into the *mss2∆* background to establish the corresponding gene overexpression strains.

### Colony morphology and *in vitro* invasion assays

*C. albicans* cells were cultured in 3 mL YPD medium overnight. The cells were washed three times with PBS. Washed *C. albicans* cells were prepared from suspensions with an OD_600_ of 1.0. A 2 µL aliquot of each dilution was applied to RPMI 1640 agar supplemented with 1 mM H_2_O_2_, 5 mM H_2_O_2_, 200 μM glutathione (GSH), 1 mM H_2_O_2_ plus 200 μM GSH and 5 mM H_2_O_2_ plus 200 μM GSH. Plates were incubated at 30°C for 2–4 days, after which images were recorded. Colony hyphal invasion depth into the agar was quantified using ImageJ by excising the colonies from the agar, acquiring images, and measuring the distance from the agar surface to the furthest hyphal growth to determine the depth of invasion [36]. The overexpression system was induced by a 50 μg/mL doxycycline-containing agar plate. Specifically, an overnight culture was adjusted to an OD_600_ of 1.0, then the cells were applied onto an agar plate supplemented with or without 50 μg/mL doxycycline. The plates were subsequently incubated at 30°C for two days to allow for colony formation and observation.

### Hyphal induction assay

*C. albicans* cells were cultured in 3 mL YPD medium overnight. The cells were washed three times with PBS. The washed *C. albicans* cells were adjusted to an OD_600_ of 0.5 and grown in RPMI 1640 medium at 30°C for 24 h. Hyphal development of the *C. albicans* cells was quantified with an Eclipse Ti inverted microscope (Nikon Instruments Inc., Melville, NY, USA).

### Cytosolic calcium concentration measurement

Calcium concentrations were measured using the calcium-sensitive dye Fluo-3 AM, as described by Chen et al. (2023). *C. albicans* cells were harvested by centrifugation and washed twice with Krebs buffer (pH 7.2), containing 132 mM NaCl, 4 mM KCl, 1.4 mM MgCl_2_, 6 mM glucose, 10 mM HEPES, 10 mM NaHCO_3_, and 1 mM CaCl_2_. The cells were incubated at 37°C for 30 min with a mixture of 5 µM Fluo-3 AM, 1% bovine serum albumin, and 0.01% Pluronic F-127 in Krebs buffer. After staining, the cells were centrifuged and incubated in calcium-free Krebs buffer at 37°C for 30 min. This step was repeated twice. Finally, the cells were washed three times with PBS and resuspended in PBS. The fluorescence intensities of Fluo-3 AM (λemission = 526 nm) were measured using a Cytek® Aurora flow cytometer (Cytek Biosciences).

### Measurement of cellular ROS

Cellular ROS was measured by fluorescent dyes H_2_DCFDA, following the protocol adapted from Chen et al. (2023). *C. albicans* cells were cultured overnight in YPD medium at 30°C. The cells were then subcultured into fresh YPD and incubated for 4 h. Cells were harvested and washed twice with PBS. Cells were adjusted to an OD_600_ of 1.0, mixed with 20 µg/mL H_2_DCFDA or H_2_DCFDA plus 150 mM H_2_O_2_ for the positive control, and incubated at 30°C for 30 min in the dark. After incubation, the cells were washed twice with cold PBS and resuspended in PBS. Fluorescence intensities were measured using a FACSCanto™ II Flow Cytometer (BD Biosciences).

### RNA isolation and RNA sequencing

Performing RNA extraction and RNA-Seq followed the established protocol [37]. Hyphae grown in RPMI 1640 liquid medium or on RPMI 1640 agar plate at 30°C. Cells were harvested after 24 h and washed 3 times with sterile water. Total RNA was extracted using the MasterPure™ Yeast RNA Purification Kit (Epicenter, Madison, WI, USA). To eliminate genomic DNA contamination, the RNA samples were treated with DNase I (Thermo Fisher Scientific, Waltham, MA, USA). RNA quality and concentration were confirmed using a BioAnalyzer for library preparation. RNA sequencing was performed by Genomics BioTech using the NovaSeq 6000 platform. Differentially expressed (DE) genes were identified using the DESeq2 statistical R package [38]. Genes with count values below 10 across all sample replicates were filtered out. Filtered counts were used to quantify log2 fold-changes and false discovery rate adjusted p-values (padj). DE genes were obtained by filtering genes with padj ≤0.05 and log2(fold-change)| ≥ 1.5. Gene counts were log-converted using the “rlog” function in DESeq2. rlog values of DE genes were used to generate heatmaps using the “pheatmap” package in R (https://github.com/raivokolde/pheatmap). Volcano plots were made using the “ggplot2” package in R. Transcriptomic data generated from this study have been deposited to the NCBI Gene Expression Omnibus (GEO) and are available under the accession number GSE295023.”

### Real-time RT-PCR assay (qRT-PCR)

The assay was conducted following the protocol established previously [37]. After RNA isolation, cDNA was synthesized from RNA using the iScript™ cDNA Synthesis Kit (Bio-Rad Laboratories, Inc., CA, USA). The reaction mixture is initially incubated at 25°C for 5 min to allow for efficient primer annealing to the RNA template. Following this, the temperature is raised to 46°C for 20 min, providing the optimal conditions for the reverse transcriptase enzyme to synthesize the complementary DNA strand. Finally, the reaction is briefly heated to 95°C for 1 min to inactivate the reverse transcriptase, preparing the newly synthesized cDNA for subsequent molecular applications. Quantitative PCR was conducted using the Bio-Rad CFX Manager (Bio-Rad Laboratories, Hercules, CA, USA) using primer pairs 541/542, 2093/2094, 2823/2824, 2821/2822, and 2825/2826 to detect *ACT1*, *UME6*, *SAC1*, *RIM8* and *ORF19.1841* expression, respectively. The experiments were independently repeated three times and statistical analysis was performed using Student’s t-test. Gene expression was normalized to the expression of the *ACT1* gene.

### Cell growth assay

Overnight cultures of *C. albicans* cells were adjusted to an OD_600_ of 0.01 using a fresh YPD medium. The cells were incubated at 30°C for 24 h with shaking. OD_600_ measurements were taken every 2 h using an Epoch 2 Microplate Reader (BioTek Instruments, Inc.) to monitor cell growth.

### Mitochondrial colocalization and morphology assay

*C. albicans* cells were cultured overnight in YPD medium at 30°C. The cells were then subcultured into fresh YPD and incubated for 4 h. MitoTracker™ Red CMXRos (200 nM) was added to the culture for mitochondria labeling, and cells were incubated at 37°C for 30 min [39]. After incubation, cells were harvested by centrifugation, washed twice with PBS, and prepared for imaging.

### Mitochondrial activity assessed by Seahorse XFe 24

Oxygen consumption rate (OCR) was measured using a Seahorse XFe 24 analyzer, following the previous protocol with slight modifications [10]. Overnight cultures of *C. albicans* cells were washed three times with PBS, transferred to 24-well XFe microplates (Seahorse Bioscience) coated with 50 μg/mL poly-D-lysine, and incubated for 1 h at 30°C to allow cell adhesion. Each well was filled with a final volume of 675 μL RPMI 1640 medium before analysis. OCR was measured for each sample at 9-min intervals using a Seahorse XFe 24 analyzer. Mitochondrial respiration was initially induced by the RPMI 1640 medium, followed by sequential injections of 100 μM triethyltin bromide (TET), 5 μM FCCP, and 2 μM antimycin A. Each experiment included four biological replicates per strain. Student’s t-test was used for statistical analyses.

### Cell invasive assay

This experiment followed the previously established protocol with minor modifications [10,40]. HeLa cells (human epithelial cells from a fatal cervical adenocarcinoma) were cultured in DMEM (Dulbecco’s Modified Eagle Medium, Thermo Fisher) supplemented with 10% fetal bovine serum (FBS) and 10 μg/mL streptomycin and 100 U/mL penicillin, and incubated at 37°C with 5% CO_2_. Overnight HeLa cell cultures on glass coverslips were washed with PBS, then co-incubated with *C. albicans* cells at 37°C with 5% CO_2_ for 1.5 h. After incubation, samples were fixed with cold 4% paraformaldehyde for 30 min. 25 μg/mL of cell non-permeant concanavalin A – fluorescein conjugate (Invitrogen) in PBS was added, and the samples were incubated for 45 min to stain extracellular *C. albicans* cells. HeLa cell membranes were then permeabilized with 0.1% Triton X-100 for 15 min. Extracellular and intracellular *C. albicans* cells were stained with Calcofluor white (10 μg/mL, 0.1 M Tris-HCl) for 20 min. Samples on coverslips were washed three times with PBS and mounted with 100% glycerol. Fluorescence of each sample was determined using an Eclipse Ti inverted microscope. Three independent experiments were conducted, with a total of at least 300 *C. albicans* cells analyzed. The percentage of *C. albicans* cells that invaded the epithelial cells was then calculated.

### Fungal virulence and host burden evaluation

Five-week-old male ICR mice were purchased from BioLASCO (Taiwan) and were used in experiments at six weeks (25–30 g) of age (*n* = 9). YPD Overnight cultures of the *C. albicans* wild-type or mutant cells were subcultured into fresh YPD and incubated for 4 h. Cells were centrifuged and washed three times with PBS. 5 × 10^5^ cells in 200 µL *C. albicans* cells were injected into the mice via tail vein injection. The infection course was assessed for 30 days to evaluate survival. Statistical significance was determined using the log-rank test with a P-value threshold of < 0.05.

For fungal burden, 5 × 10^5^ *C. albicans* wild-type or mutant cells were injected in mice and sacrificed three days post-infection. Kidneys and spleens (*n* = 5 per group) were harvested and weighed. Organs were homogenized in 10 mL of PBS for 2 min at 30,000 rpm using an IKA T10 Basic Ultra-TURRAX® disperser (IKA, Germany). Samples were serially diluted and plated onto YPD agar supplemented with 100 µg/mL chloramphenicol. The CFUs (colony-forming units) were quantified after 2 days of incubation. All procedures involving animals were conducted in compliance with the guidelines approved by the Institutional Animal Care and Use Committee (IACUC) of National Taiwan University (Approval No. NTU-112-EL-00003). Sample sizes were based on prior literature and pilot studies assessing systemic *C. albicans* infection models [10]. 9 mice per group were used for survival analysis, allowing sufficient statistical power to detect virulence differences via log-rank testing. For fungal burden determination in kidneys and spleens, 5 mice per group were used. Mice were euthanized using CO_2_ inhalation in a controlled manner, adhering to the AVMA Guidelines for the Euthanasia of Animals. The CO_2_ was supplied from a compressed gas cylinder, and its flow rate was precisely regulated using a pressure regulator and flowmeter. To ensure complete euthanasia, CO_2_ exposure was maintained for at least 1 min after respiratory arrest, or for a sufficient duration to confirm death. Inclusion and exclusion criteria were established a priori. Animals that failed to meet the predefined experimental conditions, such as unsuccessful colonization, unrelated health abnormalities, or unexpected mortality,were excluded from further analysis. All other experimental units and data points were retained for final analysis. We confirm that our study adhered to the ARRIVE guidelines. A completed ARRIVE checklist is included in the supplementary materials.

### Statistical analyses and study period

Data are presented as mean ± standard deviation (SD). The Shapiro – Wilk test was used to assess the normality of data distributions. Student’s *t*-tests were used with a 95% confidence level if distributions were normal. For datasets that did not meet normality assumptions, the non-parametric Mann – Whitney U test was applied. Survival analyses in virulence assays were evaluated using the log-rank test, with statistical significance defined as *p* < 0.05. All statistical evaluations and graph visualizations were performed using Excel or GraphPad Prism 10 (GraphPad Software, Inc.). All *in vitro* and *in vivo* assays and transcriptomic experiments were performed from March 2022 to June 2025.

## Results

### Contact-dependent hyphal development requires mitochondrial integrity mediated by *MSS2*

**Figure 1.**
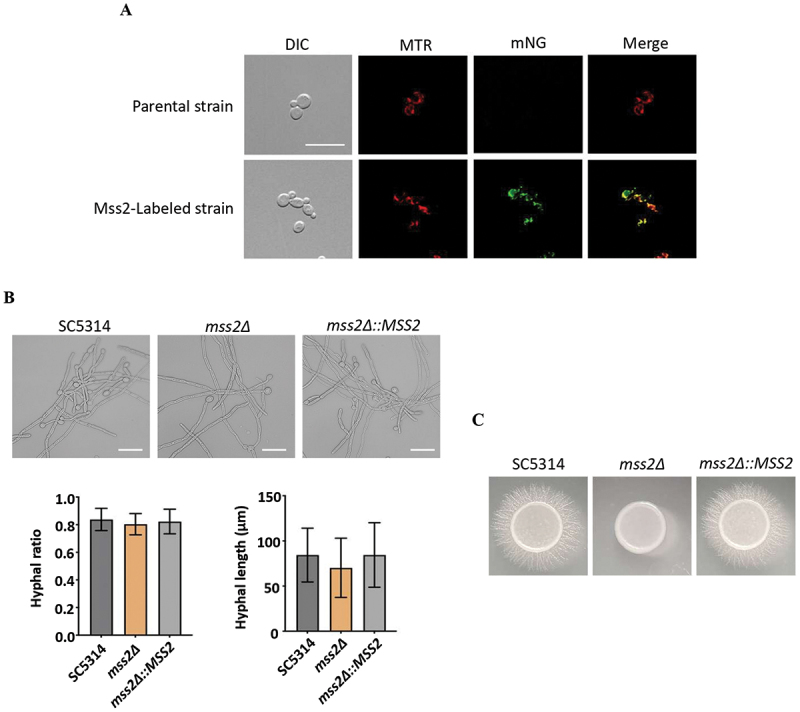
Mss2 localizes to mitochondria and is required for invasive growth in *C. albicans*. (A) Subcellular localization of Mss2-mNeongreen in *C. albicans* SC5314. Fluorescence microscopy images showed that Mss2-mNeongreen co-localizes with the mitochondrial marker MitoTracker Red, indicating mitochondrial localization of Mss2. (B) Hyphal development of wild-type (SC5314), *mss2Δ* and complementary strains under RPMI 1640 liquid culture conditions. Hyphal morphology was evaluated microscopically, with 100 cells analyzed per replicate across three independent experiments. Statistical comparisons were made using unpaired, two-tailed Student’s *t*-tests. Scale bar: 20 μm. Upper panel: Representative images of each strain. Lower panel: quantitative results of hyphal ratios and hyphal length of each strain. (C) The WT, *mss2Δ* and complemented strains were spotted onto RPMI 1640 solid agar and colony morphology was photographed after 5 days incubation at 37°C.

Previously, we demonstrated that a mitochondrial-associated gene, *MSS2*, is crucial for ATP production and mitochondrial function in *C. albicans* [10]. To determine the subcellular localization of the Mss2 protein, an SC5314 strain expressing Mss2-mNeonGreen was constructed and signal found to localize to the mitochondria (Figure 1(A)). Several studies have shown that mitochondrial dysfunction disrupts virulence-associated functions, including the yeast-hyphal transition, biofilm development, and cell wall integrity [39,41–44]. Consistent with the previous report [10], *MSS2* was dispensable for hyphal development in suspension but critical for invasive growth on solid agar (Figure 1(B,C)).

**Figure 2.**
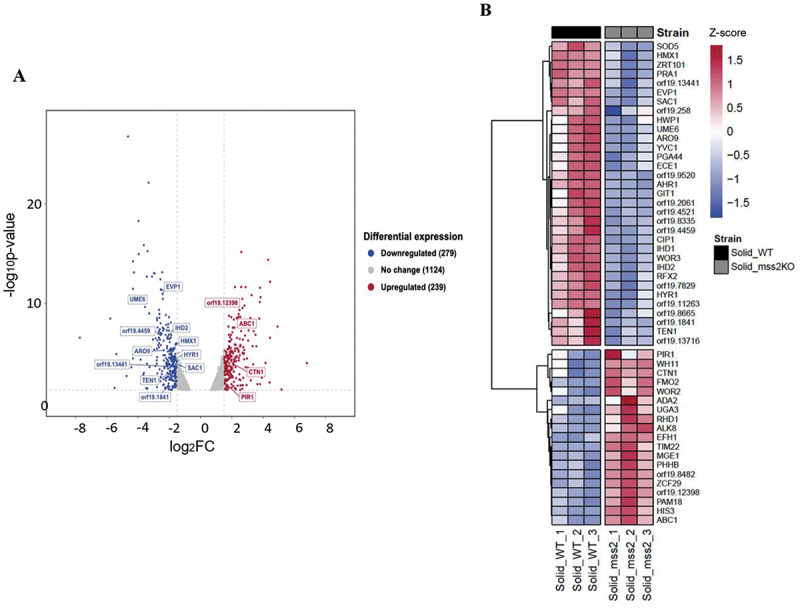
RNA-Seq analysis of the *C. albicans mss2Δ* reveals contact-specific gene expression changes. (A) Volcano plot illustrating the distribution of gene expression changes. Each dot represents a gene; the x-axis indicates log_2_ Fold change, and the y-axis represents log_10_. Genes with statistically significant (log2FC≥1.5 or ≤-1.5; *p* < 0.05) upregulation (red) and downregulation (blue) are highlighted. (B) The heat map displays clusters of selected genes significantly upregulated (red) or downregulated (blue) in the *mss2Δ* compared to the wild-type (WT) when grown on solid medium. Expression levels were normalized and hierarchically clustered.

**Figure 3.**
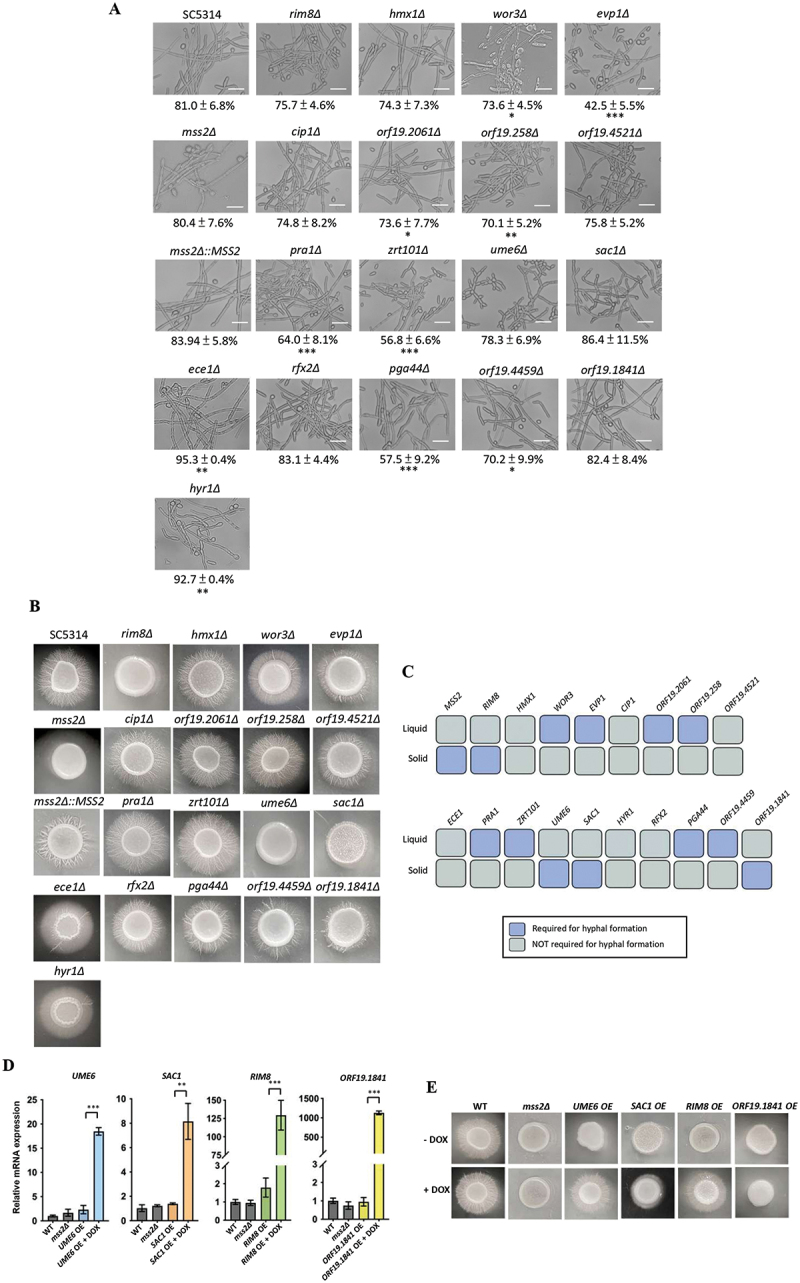
Evaluation of *MSS2* and downstream target genes for hyphal morphogenesis under both liquid and solid growth conditions. (A) Quantitative analyses of hyphal development in RPMI 1640 liquid medium of *MSS2* downstream gene mutants. Bottom panel, quantification of the number of hyphae. Values are the mean ± SD from three independent replicates (100 cells/per replicate) quantified microscopically. “*” represents *p* < 0.05 and “***” represents *p* < 0.001 for the difference with the wild-type (SC5314) strain. (B) Images of invasive hyphal growth of Mss2-regulated gene deletion strains on RPMI 1640 solid agar. (C) Summary of hyphal morphologies in the *C. albicans mss2Δ* and its downstream gene mutants under liquid and solid culture conditions. Scale bar: 20 μm. (D) Quantitative PCR analysis showed that the overexpression of *UME6*, *SAC1*, *RIM8*, and *ORF19.1841* was significantly increased in the *mss2Δ* strain upon doxycycline induction. Data from the overexpression strains were compared under induced (+DOX) and non-induced (–DOX) conditions. Values represent the mean ± SD from three independent experiments. “**” represents *p* < 0.01 and “***” represents *p* < 0.001. (E) Images of hyphal growth for gene overexpression strains in an *MSS2* knockout background on RPMI 1640 solid agar.

To determine how *MSS2* governs invasive hyphal growth on solid surfaces, cells were grown in liquid or solid media and subjected to RNA-sequencing (RNA-Seq). Differentially expressed genes were filtered based on a log2FC ≥ 1.5 or ≤-1.5 and a statistical threshold of *p* < 0.05. A total of 239 genes were up-regulated and 279 genes were down-regulated in the *mss2* null mutant when cultured on the solid medium compared to the parental control (Figure S1A, Table S3 and Table S4). In contrast, 58 genes were up-regulated and 38 genes were down-regulated under liquid culture conditions (Table S4). Notably, 19 up-regulated and 16 down-regulated genes were expressed under both solid and liquid conditions, indicating a core set of Mss2-responsive genes independent of the growth environment (Table S4). These overlapping genes were excluded from subsequent analyses, allowing us to narrow down the genes that were specifically influenced by *MSS2* deletion during growth on solid media. We further selected genes associated with hyphal formation, biofilm development, mitochondrial function, and other candidates of interest for further investigation (Figure 2(A,B)). Mutants were constructed and hyphal induction assays were performed in both liquid and solid media (Figure 3(A,B)). We found that deletion of *SAC1*, *RIM8*, and *ORF19.1841* exhibited comparable phenotypes to those of the *mss2Δ* mutant, in which a significant defect in hyphal formation was observed on solid agar but not in liquid culture (Figure 3(A,B,C)). Interestingly, although the hyphae ratios formed by *ume6Δ* mutants were comparable to those of *mss2Δ* cells in liquid medium, deletion of *UME6* resulted in the formation of short and branched pseudohyphae that were distinct from those observed in control cells or *mss2Δ* cells under these conditions (Figure 3(A)). Furthermore, qPCR analyses revealed that Mss2 regulates the expression of *UME6*, *SAC1*, *RIM8*, and *ORF19.1841* when cells were cultured on solid media (Figure S1B and S1C). To investigate the roles of these genes in *MSS2*-mediated invasive hyphal growth, we constructed overexpression (OE) strains of these genes in the *mss2Δ* strain and compared their phenotypes with those of the parental *mss2Δ* and wild-type strains. Quantitative PCR analysis demonstrated that overexpression of *UME6*, *SAC1*, *RIM8*, and *ORF19.1841* was significantly increased in the *mss2Δ* strain upon doxycycline (Dox+) induction (Figure 3(D)). Furthermore, the *UME6*, *RIM8* or *SAC1* OE strain in a *mss2Δ* strain is able to form hyphae and partially recover the phenotype on the solid agar media, compared to the wild-type strain, while the *ORF19.1841* OE strain did not (Figure 3(E)). This finding illustrates that *UME6*, *RIM8*, and *SAC1*, but not *ORF19.1841*, play a more key role in *MSS2*-mediated invasive hyphal growth.

### Mitochondrial phenotypes of *mss2Δ* cells and other hyphal regulator mutants

**Figure 4.**
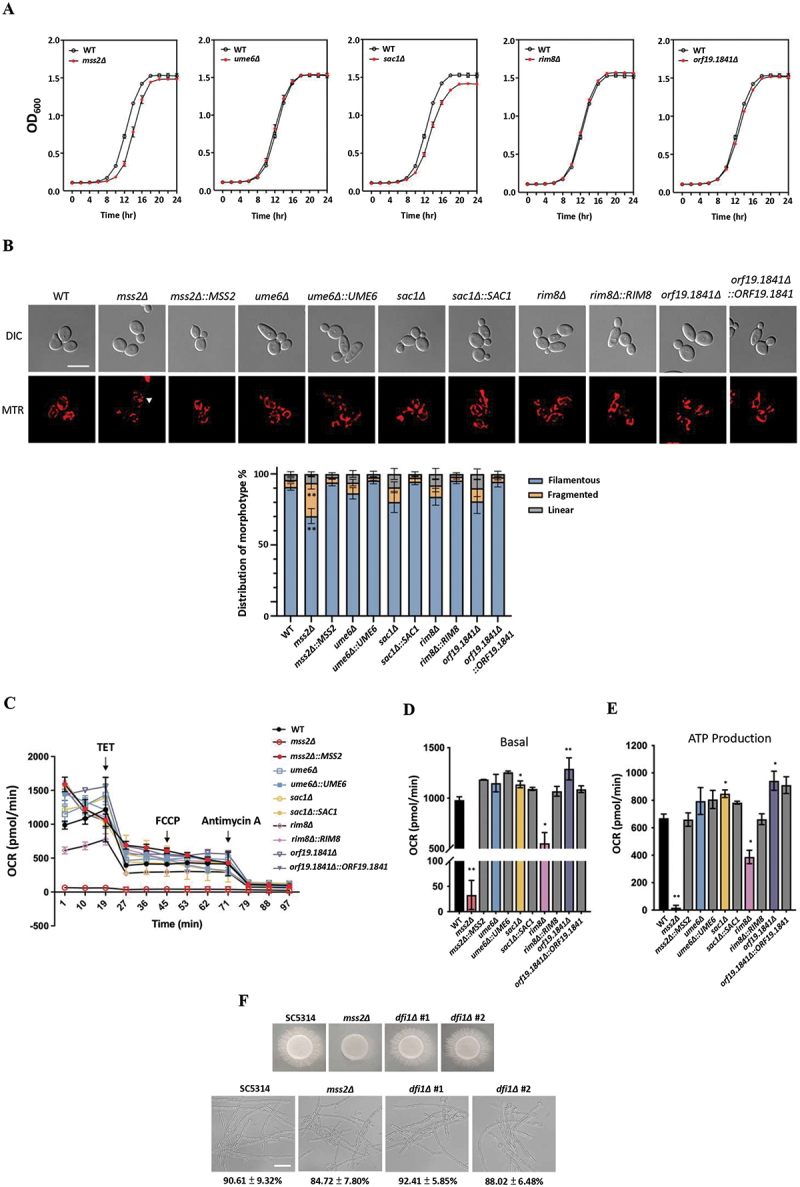
Mitochondrial function of *mss2Δ*, *ume6Δ*, *sac1Δ*, *rim8Δ* and *orf19.1841Δ* cells and the role of *DFI1* in invasive hyphal growth. (A) Growth rates of *C. albicans* wild-type (WT) and mutant strains in YPD liquid medium. (B) Analysis of *mss2Δ*, *ume6Δ*, *sac1Δ*, *rim8Δ* and *orf19.1841Δ* cells for mitochondrial phenotypes. Values are the mean ± SD from three independent replicates (300 cells/per replicate).“*” represents *p* < 0.05 and “***” represents *p* < 0.001 for the difference with the wild-type (WT) strain. Left panel: Representative images of mitochondrial morphology visualized using MitoTracker staining. Right panel: Quantitative results of mitochondrial morphology of each strain. (C) Mitochondrial respiration activity in *C. albicans* was tested using the Seahorse Analyzer. The assays were conducted by measuring the oxygen consumption rate (OCR) after sequential injections of 100 μM triethyltin bromide (TET), 5 μM FCCP, and 2 μM antimycin A. Quantification of respiratory activity under (D) basal and (E) ATP production conditions, with reference to the mitochondrial respiration data obtained in C. Values are the mean ± SD from three independent replicates. “*” represents *p* < 0.05 and “**” represents *p* < 0.01 for the difference with the WT strain. (F) Comparison of strains for invasive growth under RPMI 1640 agar and liquid media. Values represent the mean ± SD from three independent experiments, each analyzing 100 cells microscopically. Scale bar: 20 μm. Top panel: Images showing hyphal growth on solid medium for each strain. Bottom panel: Representative images of hyphae in liquid medium for each strain. The quantitative results of hyphae and pesudohyphae ratios are shown below.

We next addressed if the genes regulated by *MSS2,* including *SAC1*, *RIM8*, *UME6*, and *ORF19.1841* are involved in mitochondrial function/morphology, cell growth, and respiration. No significant differences in cell growth rates were detected between the wild-type control and the mutant strains (Figure 4(A)). Notably, however, the *mss2Δ* mutant displayed a higher level of fragmented mitochondria compared with the wild-type control and the other mutants and all the complementary strains recovered the phenotypes (Figure 4(B)). Although no studies in *C. albicans* have linked *MSS2* to mitochondrial structure, studies in *S. cerevisiae* Mss2 (ScMss2) have shown that ScMss2 interacts with Cox18 and Pnt1, and that this partnership is essential for correctly exporting the Cox2 C-terminus for mitochondrial maintenance [45–47]. Thus, the *C. albicans* Mss2 might interact with other mitochondrial subunits and contribute to mitochondrial structural stability in *C. albicans*, as its deletion would perturb inner-membrane protein insertion and respiratory‐chain organization, plausibly explaining the structural defects we observed. Consistent with a previous report [10], *mss2Δ* cells also displayed severe defects in basal respiration and ATP production compared to wild-type cells or other mutant cells (Figure 4(C–E)). Interestingly, *rim8Δ* cells also exhibited a mild but significant respiration defect, while *sac1Δ* and *orf19.1841Δ* strains actually showed slightly increased basal respiration and ATP production rates (Figure 4(C–E)). These findings indicate that although Mss2 plays a critical role in mitochondrial function, the observed effects in other mutants suggest a multifactorial regulatory network.

Dfi1 has previously been shown to be involved in contact-dependent invasive growth [16,17]. However, RNA-Seq analysis revealed no significant differences in the expression of *DFI1* between wild-type and *mss2Δ* cells. To further compare the roles of *MSS2* and *DFI1* in invasive growth, *dfi1Δ* strains were constructed and found to exhibit normal invasive growth phenotype on RPMI 1640 solid medium (Figure 4(F)). These findings indicate that *DFI1* is not essential for invasive growth under the conditions tested here, and that Mss2 mediates invasive hyphal growth by a mechanism that is independent of Dfi1 function.

### *MSS2* modulates invasive growth through ROS-mediated regulation of gene expression

**Figure 5.**
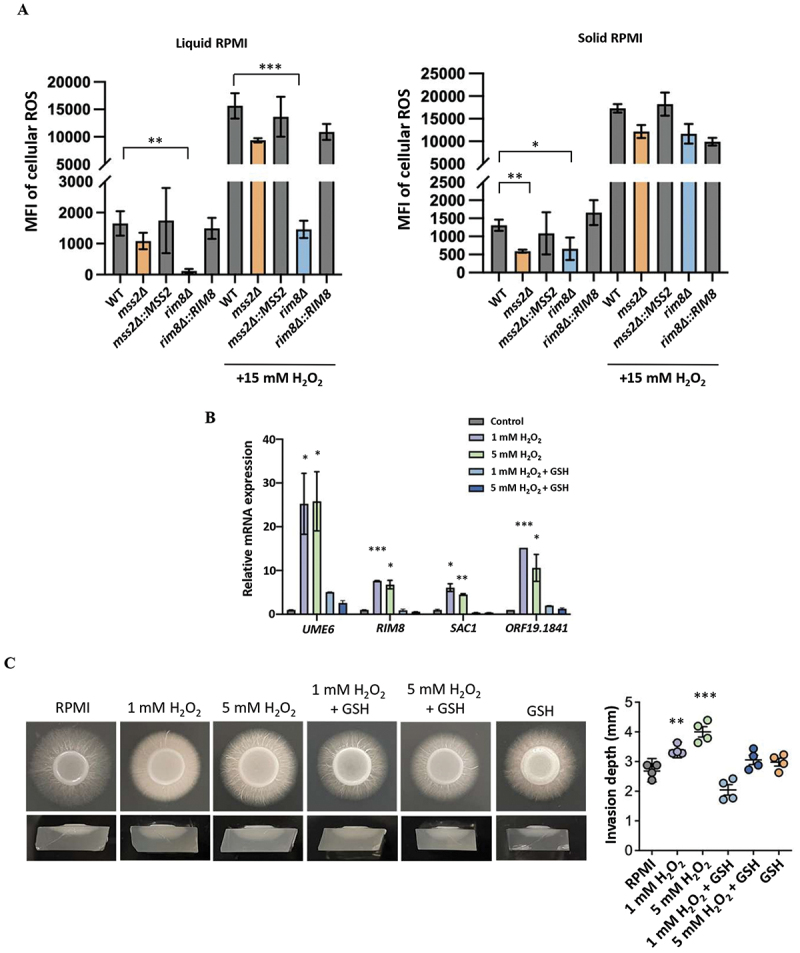
ROS contribute to *MSS2*-regulated gene expression and invasive hyphal development of *C. albicans*. (A) Analysis of ROS production under solid growth and liquid culture conditions. ROS levels were measured under liquid and solid RPMI 1640 culture conditions using H_2_DCFDA staining and flow cytometry (MFI: Mean Fluorescence Intensity). (B) RT-qPCR analysis of Mss2-regulated genes (*UME6*, *SAC1*, *RIM8*, and *ORF19.1841*) with or without H_2_O_2_ or the antioxidant glutathione (GSH). (C) Invasive hyphal growth of WT cells on RPMI 1640 agar in the presence and absence of H_2_O_2_. Right panel: Quantitative analyses of invasion depth with or without ROS/GSH. Values represent the mean ± SD from three independent experiments. “**” represents *p* < 0.01 and “***” represents *p* < 0.001 for the difference with the untreated group.

Gene Ontology (GO) and Kyoto Encyclopedia of Genes and Genomes (KEGG) pathway analyses of Mss2-regulated genes did not reveal any specific or significantly enriched signaling pathways. In addition, there are no known functional or regulatory connections between Ume6, Rim8, Sac1, and Orf19.1841, and the mitochondrial phenotypes associated with each of these mutant strains are divergent. We explored the possibility that Mss2 regulates these genes via its role in mitochondrial respiration and ROS production, as the latter can act as secondary signaling molecules to regulate gene expression and modulate host-fungal pathogen interactions [41,43,48,49]. Exogenous ROS, such as hydrogen peroxide (H_2_O_2_), have also been shown to induce filamentation in *C. albicans* [50]. Here, cellular ROS levels were quantified using a H_2_DCFDA probe and flow cytometry during growth on solid media. *mss2Δ* and *rim8Δ* strains were tested due to their mitochondrial dysfunction in basal respiration and ATP production rates (Figure 4(C–E)). Results showed that the *mss2Δ* mutant exhibited a significant reduction in ROS production relative to the wild-type control when grown on solid agar but not during liquid culture (Figure 5(A)). Interestingly, the *rim8Δ* mutant showed significant reductions in ROS production in both liquid and solid culture conditions (Figure 5(A)), further demonstrating that mitochondrial dysfunction impacts ROS production in *C. albicans*. Furthermore, the *rim8Δ* mutant showed a significant reduction in ROS levels even after H_2_O_2_ treatment in liquid medium (Figure 5(A)), suggesting that Rim8 may be associated with antioxidant regulation. RT-qPCR analysis also revealed that the set of *MSS2*-dependent genes, including *SAC1*, *RIM8*, *UME6*, and *ORF19.1841*, were upregulated after challenging cells with exogenous ROS (H_2_O_2_), whereas co-treatment with the reducing agent glutathione (GSH) quenched the increased expression of these genes (Figure 5(B)). Moreover, ROS treatment promoted filamentation and enhanced the invasive ability of wild-type cells on RPMI 1640 agar (Figure 5(C)), while the addition of the exogenous antioxidant GSH effectively blocked the effects of ROS treatment on invasive capacity (Figure 5(C)). Collectively, these findings indicate that *MSS2* modulates hyphal formation and invasive growth, at least in part, through alterations in ROS-mediated regulation of downstream target genes.

### *MSS2* modulates downstream target genes through a dynamic calcium-ROS interplay

**Figure 6.**
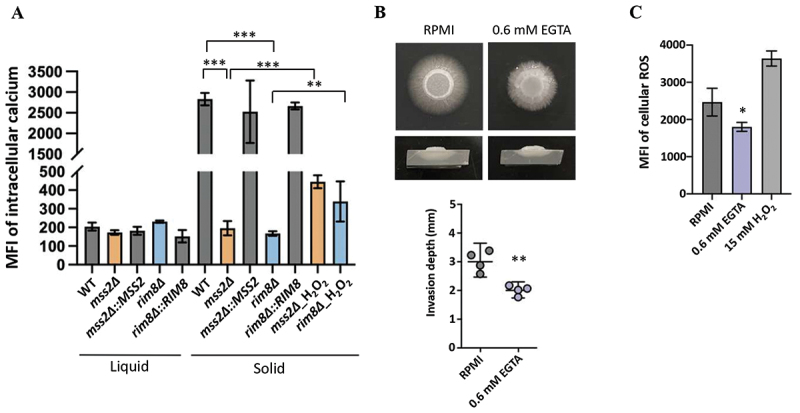
Mss2 regulates calcium and ROS during invasive hyphal growth in *C. albicans*. (A) Calcium levels were measured in WT and *mss2Δ* cells grown under liquid or solid RPMI 1640 culture conditions using the calcium indicator Fluo-3 am and subjected to flow cytometry. (B) The *C. albicans* WT cultured exhibited impaired invasive hyphal growth on RPMI 1640 agar supplemented with EGTA. Top panel: Representative images of hyphal growth and invasion ability. Bottom panel: Quantitative analyses of invasion depth with or without EGTA. (C) Intracellular ROS levels in WT cells under solid growth conditions with or without EGTA or H_2_O_2_. Values represent the mean ± SD from three independent experiments. “*” represents *p* < 0.05; “**” represents *p* < 0.01 and “***” represents *p* < 0.001 for the difference with the untreated group.

ROS have been closely associated with regulating intracellular calcium homeostasis [24]. Both ROS and calcium play critical roles as second messengers that are involved in intercellular signaling networks in mammalian cells [26]. In *C. albicans*, calcium accumulation is important for the promotion of invasive hyphal growth [1,20,51]. Cytosolic calcium levels were measured in wild-type and *mss2Δ* and *rim8Δ* strains under liquid and solid culture conditions. Results showed that calcium levels in wild-type cells were significantly elevated when grown on solid medium compared to liquid medium, and that treatment with the calcium chelator EGTA caused a significant reduction in calcium levels on solid agar (Figure 6(A)). In contrast, *mss2Δ* and *rim8Δ* cells exhibited substantially lower calcium concentrations on solid media, but not in the liquid culture condition, compared to the wild-type strain. Interestingly, exogenous ROS (H_2_O_2_) significantly increased intracellular calcium levels in the *mss2Δ* and *rim8Δ* mutants (Figure 6(A)), indicating that calcium-ROS imbalance profoundly impacts the invasive hyphal formation. Given that calcium has been implicated in thigmotropic behavior of *C. albicans* on solid surfaces [1,20,51], we further assessed the role of calcium in invasive growth in *C. albicans* grown on RPMI 1640 agar with or without EGTA supplementation. As shown in Figure 6(B), wild-type cells exhibited significant defects in filamentation and invasive capacity. Moreover, ROS production was markedly reduced in EGTA-treated *C. albicans* cells on solid medium (Figure 6(C)). These findings suggest that dysregulation of calcium-ROS signaling in the *mss2Δ* mutant, particularly under solid growth conditions, explains its impaired filamentation and invasive growth.

### Mss2 target genes are involved in invasive hyphal growth *in vitro* and *ex vivo*

**Figure 7.**
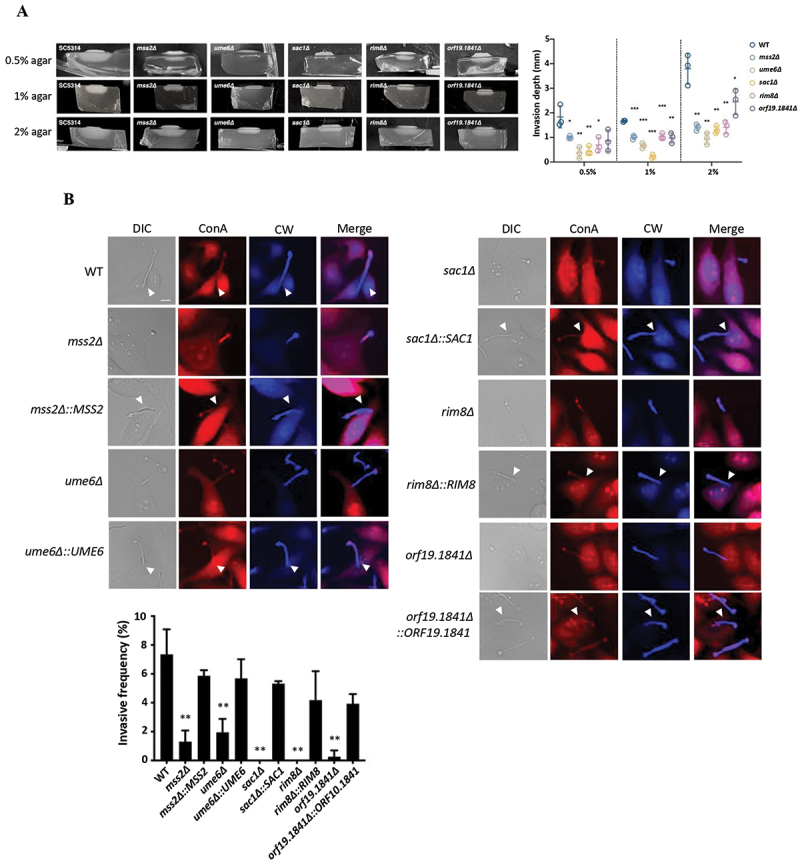
*MSS2* and its downstream genes are required for invasive growth on solid agar and into host epithelial cells. (A) Agar invasion assays were performed on RPMI 1640 plates containing 0.5% to 2% agar to evaluate the depth of hyphal penetration. Right panel: Representative images of hyphal growth and invasion ability. Left panel: Quantitative analyses of invasion depth on RPMI 1640 agar medium. (B) *Ex vivo* invasion assays were performed using human HeLa epithelial cells co-incubated with *C. albicans* strains for 1.5 h, followed by fluorescence labeling and microscopic imaging. Values represent the mean ± SD from three independent experiments. “*” represents *p* < 0.05; “**” represents *p* < 0.01 and “***” represents *p* < 0.001 for the difference with the WT strain.

Agar invasion assays demonstrated that the depth of invasion in strains deleted for Mss2 target genes (*ume6Δ*, *sac1Δ*, *rim8Δ*, and *orf19.1841Δ* strains) was significantly reduced compared to the wild-type strain using 0.5%, 1% and 2% agar plates (Figure 7(A)). These mutant strains were also examined for their ability to invade HeLa epithelial cells using a differential fluorescence labeling approach. Assays showed that *mss2Δ*, *ume6Δ*, *sac1Δ*, *rim8Δ*, and *orf19.1841Δ* strains all exhibited a significant reduction in cell invasion, with approximately 0–2% invasion rates compared to 7.3% invasion by the wild-type strain (Figure 7(B)). Mutant strains that were complemented for the deleted gene showed a restored invasion ability, confirming that the phenotypes were attributable to the targeted genes (Figure 7(B)). These data establish the critical roles of *MSS2* and Mss2-regulated downstream genes (*UME6*, *SAC1*, *RIM8*, and *ORF19.1841*) in modulating invasion into both abiotic and biotic niches.

### Mss2-regulated downstream genes are required for virulence

**Figure 8.**
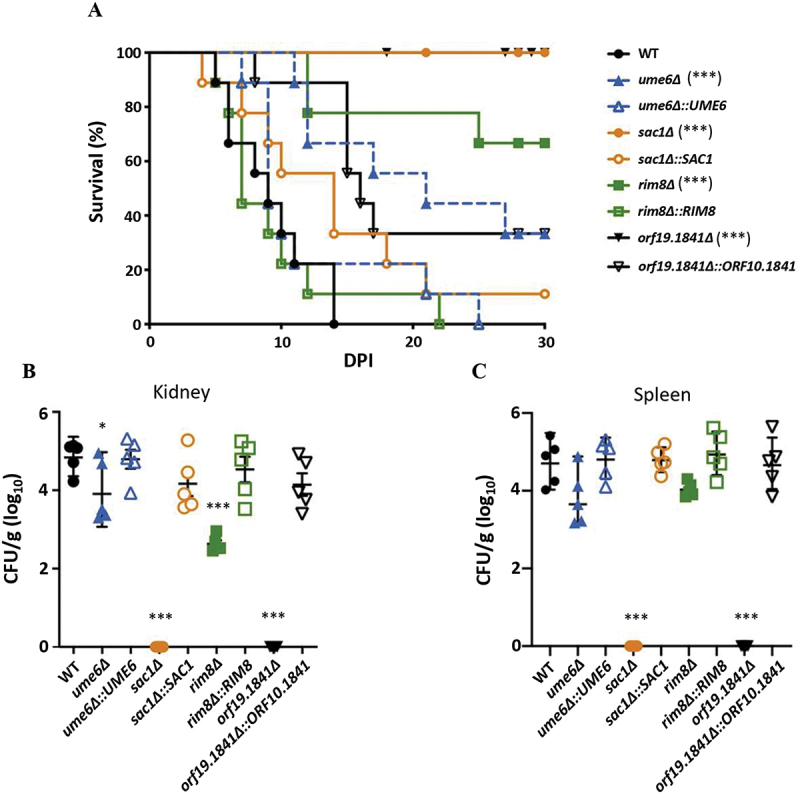
Mss2-regulated genes are required for *C. albicans* virulence (A) Survival analysis was conducted over 30 days following tail vein injection (*n* = 9). Statistical significance was determined using the log-rank test. “***” represents *p* < 0.001. (B) Colony-forming units (CFUs) were determined in the kidneys of mice sacrificed 3 days after infection (C) CFU counts in the spleens of infected mice. Values represent the mean ± SD from three independent experiments. “*” represents *p* < 0.05 and “***” represents *p* < 0.001 for the difference with the WT strain.

*MSS2* is required for systemic virulence in *C. albicans* as shown using a tail vein model of murine infection [10] and we similarly evaluated *UME6*, *SAC1*, *RIM8*, and *ORF19.1841* deletion mutants in this model. Results showed that mice infected with *sac1Δ* or *orf19.1841Δ* strains exhibited 100% survival, while mice infected with *ume6Δ* or *rim8Δ* strain displayed survival rates of 33% and 66%, respectively, all of which were significantly different from the wild-type strain (Figure 8(A)). Furthermore, all mutant strains showed significantly reduced kidney fungal burdens compared to the wild-type strain (Figure 8(B)). Interestingly, spleen fungal burdens in *ume6Δ* and *rim8Δ* mutants were comparable to those in the wild-type strain (Figure 8(C)), consistent with the survival data, in which both mutants still retained some virulence. Notably, our previous study demonstrated that the survival rate of *mss2Δ*-infected mice was 88%, accompanied by a fungal burden of approximately 2–3 log_10_ CFU in the kidneys and ~1 log_10_ CFU in the spleen [10]. Collectively, these findings indicate that the Mss2-regulated genes, including *UME6*, *SAC1*, *RIM8*, and *ORF19.1841*, contribute substantially to *C. albicans* virulence, exhibiting trends consistent with those observed in the *mss2Δ* mutant [10].

## Discussion

Beyond their well-known role in energy production, mitochondria are crucial regulators of cellular signaling through the generation of ROS molecules, whose dual nature allows them to cause cellular damage yet also function as essential messengers [26,48,49,52,53]. Mitochondria also orchestrate intracellular calcium signaling and homeostasis, profoundly influencing metabolism, apoptosis, and stress responses within the cell [54–56]. Compelling evidence underscores a sophisticated interplay among Ca^2+^ ions, ROS, and mitochondria, forming a nexus that governs critical pathological processes in cancer and neurodegenerative diseases [25,30,31]. For example, in cancer, excessive mitochondrial Ca^2+^ uptake amplifies ROS production, activating signaling cascades that potentiate tumor invasion and metastasis [29,30]. Similarly, in neurodegenerative conditions such as Alzheimer’s and Parkinson’s, disrupted mitochondrial Ca^2+^ and ROS homeostasis precipitates neuronal dysfunction and apoptotic cell death, emphasizing their integral role in the molecular etiology of these disorders [31,57,58].

In fungal pathogens, mitochondria also represent a key hub for ROS generation and calcium homeostasis and this interplay is important for cellular function, adaptation, pathogenicity, and survival [24,59]. Dysregulated mitochondrial Ca^2+^ uptake enhances ROS accumulation, influencing critical processes such as morphogenesis, metabolic reprogramming, and virulence [22,50,60,61]. Furthermore, antifungal agents such as sophorolipid, LL-37, and naringin can disrupt mitochondrial function by elevating ROS and disturbing the calcium balance of *C. albicans* [62–64]. These findings highlight mitochondria as a central hub integrating ROS and calcium signaling to govern fungal viability. *C. albicans* genes such as *NDH51*, *ATP2, GOA1*, *FZO1*, *NUO1* and *NUO2* govern mitochondrial function and dynamics, impacting hyphal formation and virulence [43,65–68]. Mitochondrial calcium signaling also modulates hyphal initiation through pathways including cAMP-PKA and calcineurin [21,42,69]. Despite these observations, it remained unclear whether mitochondrial dysfunction specifically impairs invasive growth on solid media without affecting hyphal formation in liquid, as seen in the *mss2Δ* mutant.

To explore this further, we investigated the genes regulated by *MSS2* and RNA-seq showed that this factor governs a broader transcriptional program on solid media than in liquid culture, regulating 518 genes versus 96 genes, respectively. Among 19 Mss2-regulated genes associated with filamentation, biofilm formation, and mitochondrial function, deletion of *UME6*, *SAC1*, *RIM8*, or *ORF19.1841* phenocopied the defect of *mss2Δ* cells, including impairing invasive growth and reducing virulence in HeLa co-cultures and systemic infection models. Interestingly, only *mss2Δ* showed marked mitochondrial fragmentation, revealing differences with the downstream effectors. Mitochondrial respiration also remained largely intact in the set of downstream mutants, although *rim8Δ* showed moderate dysfunction, again indicating that Mss2-mediated filamentation is not solely mediated by mitochondrial fitness. Interestingly, the *rim8Δ* mutant displayed reduced ROS levels under both liquid and solid growth conditions, while alterations in intracellular calcium were restricted to solid medium. By contrast, the *mss2Δ* mutant exhibited concurrent reductions in both ROS and calcium specifically under solid culture. Nevertheless, the convergence of both mutants indicates that disruption of calcium–ROS homeostasis is a key determinant impairing invasive hyphal development. Furthermore, the *rim8Δ* mutant displayed markedly reduced ROS levels even after H_2_O_2_ challenge, a phenotype most evident in liquid culture but less pronounced on solid medium. This difference may reflect the more uniform exposure of individual cells to oxidative stress in liquid, whereas colony stacking on agar limits H_2_O_2_ penetration. This unexpected phenotype suggests that Rim8 may participate in pathways linked to oxidative stress responses or antioxidant regulation. The precise mechanism underlying this phenotype remains unclear, and we are currently investigating it.

In *Aspergillus fumigatus*, Cox10-mediated mitochondrial dysfunction disrupts calcium homeostasis, leading to activation of the calcineurin pathway and inducing genes involved in cell wall biosynthesis and drug resistance, thereby promoting invasive growth under antifungal stress [70]. Furthermore, hydrogen peroxide causes mitochondrial constriction and division in *A. nidulans*, and this process depends on calcium and the ERMES complex (ERM-mitochondria encounters) for the constriction and division phases [71]. In *Neurospora crassa*, staurosporine-induced cell death clearly illustrates how mitochondria, ROS, and Ca^2+^ are interconnected. Proper function of mitochondrial complex I and the dehydrogenase Nde1 is needed to produce the ROS that trigger Ca^2+^ signals [72]. This shows that mitochondrial activity controls ROS levels, and these ROS in turn drive Ca^2+^ dynamics during stress. These results may illuminate how mitochondrial function, calcium signaling, and oxidative stress converge to shape fungal physiology and pathogenesis. In this study, *C. albicans mss2Δ* with compromised mitochondrial function, exhibited phenotypes with disrupted invasive growth on solid medium but normal hyphae in liquid medium. Transcriptomic analysis of *mss2Δ* cells suggested that the phenotypes may result from disrupted mitochondrial function, leading to an imbalance in intermediates such as ROS and calcium. Indeed, *mss2Δ* mutants showed reduced ROS production on solid media, correlating with defective filamentation. The phenotypic defect in filamentation could be rescued by addition of exogenous ROS. Expression of the Mss2 target genes, including *UME6*, *SAC1*, *RIM8* and *ORF19.1841*, was also highly induced in the presence of ROS. A connection between reduced cytosolic calcium levels and invasive growth defects suggests that Mss2 May modulate these processes by coordinating ROS and calcium homeostasis. Indeed, both ROS and Ca^2+^ act as secondary messengers that modulate transcriptional programs linked to hyphal morphogenesis and invasion [50,73–76]. Exogenous hydrogen peroxide is sufficient to trigger hyphal differentiation and invasive growth, underscoring that oxidative cues feed into the transcriptional network. Furthermore, Ca^2+^ and ROS signaling are known to intersect and globally remodel transcription during stress adaptation and virulence [50,73,74,76]. Although no prior studies have directly reported calcium–ROS imbalance-driven regulation of *UME6, SAC1, RIM8*, and *ORF19.1841* genes in *C. albicans*, we propose that *MSS2* disruption perturbs calcium–ROS homeostasis, which secondarily influences their expression through broader transcriptional rewiring. These interesting findings are currently under investigation to clarify the precise regulatory mechanisms and to determine why the imbalance mainly affects invasive hyphal formation on solid agar rather than in liquid medium.

Dfi1 has also been shown to regulate *C. albicans* invasive growth, and acts as a key regulator of Cek1p activation [16,17]. However, our RNA-Seq analysis revealed that *DFI1* expression was not regulated by *MSS2* and *dfi1Δ* mutants maintained the capacity to form invasive hyphae on RPMI 1640 agar, similar to the wild-type strain, but in contrast with *mss2Δ* cells. Thus, *DFI1* is not universally required for contact-dependent invasion and Mss2 must act through an alternative pathway independent of the Dfi1–Cek1 axis. This observation supports the idea that invasive growth is regulated by a context-dependent signaling network. Junier et al. (2022) showed that under low-iron conditions, invasive hyphal growth in *dfi1Δ* strains could be rescued via activation of Sef1 and Czf1, highlighting how invasive growth can be mediated by multiple pathways. The flexibility of these mechanisms is also illustrated by the loss of mitochondrial Complex I, III, or IV genes that fail to form hyphae on solid media under aerobic conditions, yet these mutants readily filament under hypoxic conditions [39]. Mitochondrial respiration is therefore critical for hyphal development in oxygen-rich environments, whereas compensatory pathways can operate under hypoxia. Together, these observations reveal that different environmental cues are able to regulate invasive growth dynamically.

**Figure 9.**
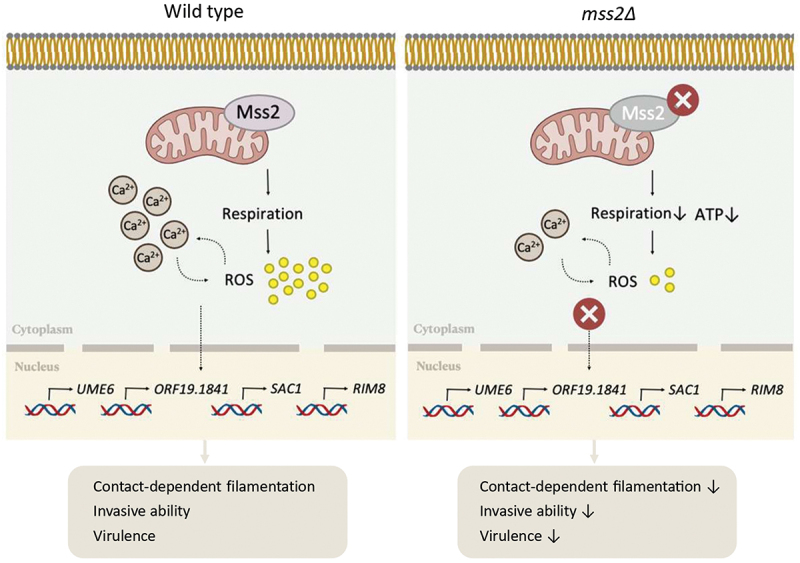
*MSS2*, a mitochondria-associated gene, regulates ROS and calcium levels required for *Candida albicans* contact-dependent invasive behavior and virulence. The Schematic model illustrates that *MSS2* is involved in mitochondrial function, which is essential for calcium and ROS homeostasis, required for downstream gene expressions and invasive hyphal growth *in vitro* and *in vivo*. Loss of *MSS2* leads to mitochondrial dysfunction, thereby causing calcium-ROS imbalance and subsequently impairing invasive growth and virulence. Illustration created using BioRender.com.

In conclusion, we demonstrate that a mitochondria-associated gene, *MSS2*, governs invasive growth and virulence via mitochondrial dependent calcium-ROS interplay (Figure 9). In this model, Mss2 promotes invasive hyphal growth by modulating calcium and ROS levels, which are the key signals for downstream target gene expression. Upon loss of *MSS2*, a calcium-ROS imbalance occurs, leading to impaired invasive growth and virulence (Figure 9). We therefore provide a previously uncharacterized connection between mitochondrial function and invasive growth in shaping *C. albicans’* pathogenic potential. Ongoing studies will address whether deficiencies in other mitochondrial components are mediated via shared disruptions in ROS and calcium signaling.

## Supplementary Material

Table S4.xlsx

Table S1.docx

Table S2.docx

Table S3.xlsx

Figure S1.tif

## Data Availability

Transcriptomic data generated from this study have been deposited in the NCBI Gene Expression Omnibus (GEO) and are available under the accession number GSE295023 (https://www.ncbi.nlm.nih.gov/geo/query/acc.cgi?acc=GSE295023. In addition, data are also included in the supplementary materials. The data that support the findings of this study are openly available in figshare at https://doi.org/10.6084/m9.figshare.29596343 [77].
