# Supplementary material for: Mss2 shapes the virulence of Candida albicans through reactive oxygen species (ROS) and calcium signaling, independent of direct transcriptional control
Source: Virulence. 2025 Nov 20;16(1):2590329. doi: 10.1080/21505594.2025.2590329 (PMC12645863; doi:10.1080/21505594.2025.2590329)
Supplement: Table S1.docx [file KVIR_A_2590329_SM4207.docx]

**Table S1. Strains used in this study.**

| **Strains** | ***MTL*** | **Genotype** | **Source** |
| --- | --- | --- | --- |
| YL2 (SC5314) | a/α | Wild-type | (1) SC5314 was originally obtained from the American Type Culture Collection (ATCC® MYA‑2876). |
| YL1700 | a/α | *mss2∆* | This study |
| YL1986 | a/α | *mss2∆::MSS2* | This study |
| YL2200 | a/α | *ume6∆* | This study |
| YL2336 | a/α | *ume6∆::UME6* | This study |
| YL2304 | a/α | *rim8∆* | This study |
| YL2355 | a/α | *rim8∆::RIM8* | This study |
| YL2322 | a/α | *sac1∆* | This study |
| YL2339 | a/α | *sac1∆::SAC1* | This study |
| YL2244 | a/α | *orf19.1841∆* | This study |
| YL2350 | a/α | *orf19.1841∆::ORF19.1841* | This study |
| YL2052 | a/α | *hyr1∆* | This study |
| YL1966 | a/α | *ece1∆* | This study |
| YL2320 | a/α | *hmx1∆* | This study |
| YL2457 | a/α | *wor3∆* | This study |
| YL2197 | a/α | *evp1∆* | This study |
| YL2279 | a/α | *cip1∆* | This study |
| YL2247 | a/α | *orf19.2061∆* | This study |
| YL2246 | a/α | *orf19.258∆* | This study |
| YL2288 | a/α | *orf19.4521∆* | This study |
| YL2226 | a/α | *pra1∆* | This study |
| YL2230 | a/α | *zrt101∆* | This study |
| YL2255 | a/α | *rfx2∆* | This study |
| YL2257 | a/α | *pga44∆* | This study |
| YL2272 | a/α | *orf19.4459∆* | This study |
| YL2416 | a/α | *dfi1∆* | This study |
| CAY16283 | a/α | pMSS2-NEON-NAT^R^ | This study |
| YL2369 | a/α | *ORF19.1841* OE in *mss2Δ* | This study |
| YL2371 | a/α | *RIM8* OE in *mss2Δ* | This study |
| YL2379 | a/α | *UME6* OE in *mss2Δ* | This study |
| CAY15777 | a/α | *SAC1* OE in *mss2Δ* | This study |

1. Bennett RJ, Johnson AD. The role of nutrient regulation and the Gpa2 protein in the mating pheromone response of *C. albicans*. Mol Microbiol. 2006;62(1):100-19.
