# Supplementary material for: Mss2 shapes the virulence of Candida albicans through reactive oxygen species (ROS) and calcium signaling, independent of direct transcriptional control
Source: Virulence. 2025 Nov 20;16(1):2590329. doi: 10.1080/21505594.2025.2590329 (PMC12645863; doi:10.1080/21505594.2025.2590329)
Supplement: Table S2.docx [file KVIR_A_2590329_SM4206.docx]

**Table S2. Oligonucleotides used in this study.**

| **Number** | **Name** | **Sequence (5’->3’)** |
| --- | --- | --- |
| 1139 | Mss2 ko 5’ flanking F | GGAGCGGGGCCCAAGTTTAACTAAAAGTGACAAG |
| 1140 | Mss2 ko 5’ flanking R | GGAGCGCTCGAGGTGTGGATACTACAACTCCATA |
| 1141 | Mss2 ko 3’ flanking F | GGAGCGCCGCGGCAATTAATTAGTTAATGCTAGG |
| 1142 | Mss2 ko 3’ flanking R | GGAGCGGAGCTCTTTACCAACACTTTATGTGGGA |
| 1710 | Mss2 ab F | GGAGCGCCGCGGGTGACAAGACGTTCACCGG |
| 1711 | Mss2 ab R | GGAGCGGAGCTCCCTTCATCGATGATAAATGGCAC |
| 2070 | Dfi1 ko 5’ flanking F | GGAGCGGGTACCGCAGGTAAAGTTGGGTGACAC |
| 2071 | Dfi1 ko 5’ flanking R | GGAGCGGGGCCCCGCCACCACCATTAGCATT |
| 2074 | Dfi1 ko 3’ flanking F | GGAGCGCCGCGGGTGGTGATTTGGCAAACTTCAAG |
| 2075 | Dfi1 ko 3’ flanking R | GGAGCGGAGCTCTATCGGACGTATCGATCGAACC |
| 2255 | Ume6 ko 5’ flanking F | GGAGCGGGGCCCGAATTTCCCGGGAGTTGTTT |
| 2256 | Ume6 ko 5’ flanking R | GGAGCGCTCGAGGAACGACTGTATGGTAATCACTT |
| 2257 | Ume6 ko 3’ flanking F | GGAGCGCCGCGGTAAGAATTAACAGGTTGACG |
| 2258 | Ume6 ko 3’ flanking R | GGAGCGGAGCTCTTAGACTTTTCCGTATGATG |
| 2554 | Ume6 ab F | GGAGCGGGGCCCCCAGAAAACTCATAAACTTCTCCGAT |
| 2555 | Ume6 ab R | GGAGCGCTCGAGCGTCAACCGTCAACCTGTTA |
| 2093 | Ume6 qPCR F | CTGGATCTGGAGTTGGGACT |
| 2094 | Ume6 qPCR R | ATGGTGTTGGTTGGGATTGT |
| 2335 | Rim8 ko 5’ flanking F | GGAGCGGGGCCCAAACGTCAACTCTCCCCTCT |
| 2336 | Rim8 ko 5’ flanking R | GGAGCGCTCGAGCTTTTGAGGCAGTTGGTGGT |
| 2337 | Rim8 ko 3’ flanking F | GGAGCGCCGCGGTTTGCAACGAAGTGACATTG |
| 2338 | Rim8 ko 3’ flanking R | GGAGCGGAGCTCCCTCCAGTTCTAGCCGTGAC |
| 2624 | Rim8 ab F | GGAGCGGGGCCCAAGAGGTGGTTTTGGTGGTG |
| 2625 | Rim8 ab R | GGAGCGCTCGAGGACGACGACGAAGACGTTATT |
| 2821 | Rim8 qPCR F | TGCCTTCCCGACAATTTTGG |
| 2822 | Rim8 qPCR R | TCTCGGTTTGGGTGGAATCA |
| 2351 | Sac1 ko 5’ flanking F | GGAGCGGGGCCCCAAGGAAAGGCGGAGTAAGA |
| 2352 | Sac1 ko 5’ flanking R | GGAGCGCTCGAGGCGTGTACGAATGGTTGAAA |
| 2353 | Sac1 ko 3’ flanking F | GGAGCGCCGCGGTTAGCTGTGGGGGTTGTTTT |
| 2354 | Sac1 ko 3’ flanking R | GGAGCGGAGCTCCAATGAAATAAGCACCCAACA |
| 2552 | Sac1 ab F | GGAGCGGGGCCCGGTGGTGATATGATCAGGGAGT |
| 2553 | Sac1 ab R | GGAGCGCTCGAGGCAGCTACAACTAAAGACAACTTTC |
| 2823 | Sac1 qPCR F | CTGGGCTGATAATGCCGAT |
| 2824 | Sac1 qPCR R | ATCTTGTCTACTACCATCAG |
| 2295 | Orf19.1841 ko 5’ flanking F | GGAGCGGGGCCCGATGGGAATCCAAATTATCG |
| 2296 | Orf19.1841ko 5’ flanking R | GGAGCGCTCGAGTTGTGATGTGTTTTCGGACA |
| 2297 | Orf19.1841ko 3’ flanking F | GGAGCGCCGCGGAAACCACTTGCTTCCCTTTT |
| 2298 | Orf19.1841ko 3’ flanking R | GGAGCGGAGCTCTGACGTGAGTCAGGATTAGG |
| 2628 | Orf19.1841 ab F | GGAGCGGGGCCCGAGCAATCTTGGGGAATAGTTGG |
| 2629 | Orf19.1841 ab R | GGAGCGCTCGAGTCGGTTATTGAAGCCGGAGA |
| 2825 | Orf19.1841 qPCR F | AGGAACCACATCAGGGAATGA |
| 2826 | Orf19.1841 qPCR R | CTCCACAATAAATTGTGCAATTGC |
| 1609 | Ece1 ko 5’ flanking F | GGAGCGGGGCCCTTTCTGGAGTAATCCTATTGTTCGC |
| 1610 | Ece1 ko 5’ flanking R | GGAGCGCTCGAGTATGTAAGATTTGTGGGCGGG |
| 1631 | Ece1 ko 3’ flanking F | GGAGCGGCGGCCGCTTTGGTGTCTCTTTGCGTGTAAA |
| 1612 | Ece1 ko 3’ flanking R | GGAGCGGAGCTCCAATCTTGTCGTGCCACTGATTA |
| 1810 | Hyr1 ko 5’ flanking F | GGAGCGGGGCCCATTCCAAGCGAGGTGAAATCAA |
| 1811 | Hyr1 ko 5’ flanking R | GGAGCGCTCGAGAGGCGTGTGATTACTTGGAATGA |
| 1812 | Hyr1 ko 3’ flanking F | GGAGCGCCGCGGGGAAACTATAAGCGGTGGTTGG |
| 1813 | Hyr1 ko 3’ flanking R | GGAGCGGAGCTCAACAGAGTACACACACTGTGTCAGGTT |
| 2407 | Hmx1 ko 5’ flanking F | GGAGCGGGGCCCCTGTGCGTGCTTTTATTCCA |
| 2408 | Hmx1 ko 5’ flanking R | GGAGCGCTCGAGTCCAACTAAAGCTTCGCAGA |
| 2409 | Hmx1 ko 3’ flanking F | GGAGCGCCGCGGTGCTCCATCTCTTCATTGGT |
| 2410 | Hmx1 ko 3’ flanking R | GGAGCGGAGCTCGTCGTTGATTGGCATTGTTG |
| 2803 | Wor3 ko 5’ flanking F | GGAGCGGGGCCCCACCTAACTCAAGTCCTCCCA |
| 2804 | Wor3 ko 5’ flanking R | GGAGCGCTCGAGGGTGGAAAGAAGAAACTGACGG |
| 2805 | Wor3 ko 3’ flanking F | GGAGCGCCGCGGCTGAGTCTCTCCCCCTTCTATTC |
| 2806 | Wor3 ko 3’ flanking R | GGAGCGGAGCTCTTTCCCCCCATCTCTGTGAA |
| 2247 | Evp1 ko 5’ flanking F | GGAGCGGGGCCCCCAATTTCATCCCCTGGATT |
| 2248 | Evp1 ko 5’ flanking R | GGAGCGCTCGAGCCAAGATGCAGCACATGAGT |
| 2249 | Evp1 ko 3’ flanking F | GGAGCGCCGCGGTTGTTTTAAGGCCAAATAGGG |
| 2250 | Evp1 ko 3’ flanking R | GGAGCGGAGCTCTCAGTTATTGGGTCCGTTCC |
| 2207 | Cip1 ko 5’ flanking F | GGAGCGGGGCCCTACGCCACAATTTCTTGGTG |
| 2208 | Cip1 ko 5’ flanking R | GGAGCGCTCGAGACCGGATAATTTTTGGCTCA |
| 2209 | Cip1 ko 3’ flanking F | GGAGCGCCGCGGTTTATGGAAATGGGGCAAGT |
| 2210 | Cip1 ko 3’ flanking R | GGAGCGGAGCTCTGCAAATAAAGCTGCAAGGA |
| 2223 | Orf19.2061 ko 5’ flanking F | GGAGCGGGGCCCTCCTGCTGCTCATGAAGTTG |
| 2224 | Orf19.2061 ko 5’ flanking R | GGAGCGCTCGAGCATGAGCCTCTGGATTCATT |
| 2225 | Orf19.2061 ko 3’ flanking F | GGAGCGCCGCGGTCTTTGTGTGCGGAATTTTG |
| 2226 | Orf19.2061 ko 3’ flanking R | GGAGCGGAGCTCCATAATGTGCGGGGGACTAA |
| 2215 | Orf19.258 ko 5’ flanking F | GGAGCGGGGCCCTCACGTGTATACCCCTCTCCA |
| 2216 | Orf19.258 ko 5’ flanking R | GGAGCGCTCGAGTCAAAATGGTGAAGAAAAACCA |
| 2217 | Orf19.258 ko 3’ flanking F | GGAGCGCCGCGGTTGCAACAAGTTGGATTTGC |
| 2218 | Orf19.258 ko 3’ flanking R | GGAGCGGAGCTCCAACCATCACCATCTGTTGC |
| 2471 | Orf19.4521 ko 5’ flanking F | GGAGCGGGGCCCGAGGAGGAGGGGACTAGGAA |
| 2472 | Orf19.4521 ko 5’ flanking R | GGAGCGCTCGAGCCCAAGCTTATGGGTATTTG |
| 2473 | Orf19.4521 ko 3’ flanking F | GGAGCGCCGCGGACTATGCGGCAAGAACATGG |
| 2474 | Orf19.4521 ko 3’ flanking R | GGAGCGGAGCTCGACGAGGAAGATGAAGATTGG |
| 2287 | Pra1 ko 5’ flanking F | GGAGCGGGGCCCTTCCGTTTTCCAAGATTCCA |
| 2288 | Pra1 ko 5’ flanking R | GGAGCGCTCGAGCAAAGGTCTTTTCCAACGAG |
| 2289 | Pra1 ko 3’ flanking F | GGAGCGCCGCGGAGTTCAGGCACCAAACAATT |
| 2290 | Pra1 ko 3’ flanking R | GGAGCGGAGCTCGGCAAATGATTGCTCCTTCT |
| 2279 | Zrt101 ko 5’ flanking F | GGAGCGGGGCCCCAAAGGTCTTTTCCAACGAG |
| 2280 | Zrt101 ko 5’ flanking R | GGAGCGCTCGAGATGTTTCCAACTATTCTGGGC |
| 2281 | Zrt101 ko 3’ flanking F | GGAGCGCCGCGGGACCACCATAATGGGTCTCG |
| 2282 | Zrt101 ko 3’ flanking R | GGAGCGGAGCTCTCAGCATGAGTCCAAGTGGT |
| 2383 | Rfx2 ko 5’ flanking F | GGAGCGGGGCCCAAGAAAAGAAAACCCCATCG |
| 2384 | Rfx2 ko 5’ flanking R | GGAGCGCTCGAGTTTGACGGCAAATGATTTTT |
| 2385 | Rfx2 ko 3’ flanking F | GGAGCGCCGCGGTGGTGGGTCTTCAATTCATT |
| 2386 | Rfx2 ko 3’ flanking R | GGAGCGGAGCTCCGGCAGCTTTTATTCCTGAT |
| 2375 | Pga44 ko 5’ flanking F | GGAGCGGGGCCCGGATTCGTTGCACTCTCTGA |
| 2376 | Pga44 ko 5’ flanking R | GGAGCGCTCGAGTGTATGATGGGGACCACGTA |
| 2377 | Pga44 ko 3’ flanking F | GGAGCGCCGCGGCCGGTAGCTAACCACAAAAC |
| 2378 | Pga44 ko 3’ flanking R | GGAGCGGAGCTCGTGGAATGTGGAGTGAAGCA |
| 2343 | Orf19.4459 ko 5’ flanking F | GGAGCGGGGCCCTGCCTTGTCTTGTCTTTTGC |
| 2344 | Orf19.4459 ko 5’ flanking R | GGAGCGCTCGAGTTGCTGGAGAATTGGGTGAT |
| 2345 | Orf19.4459 ko 3’ flanking F | GGAGCGCCGCGGCTGCCACTCCTACAGCTCCT |
| 2346 | Orf19.4459 ko 3’ flanking R | GGAGCGGAGCTCGGATGTGTGTGGATGTGGGG |
| 541 | Act1 qPCR F | TGGACTTGTGTTGTTATCTGGACT |
| 542 | Act1 qPCR R | CTTGCTGTGTTTGTGTTTGTGTTG |
| 9130 | Mss2-ORF 5_fwd BsaI | GGACCGGGTCTCTGGAGTTAATTAAATCATGGATT  TGGTGGTGG |
| 9131 | Mss2-ORF 5_rev BsaI | GGACCGGGTCTCTACAATTTTACTAGTAACTAACT  TTATATCATTCAATCTAG |
| 9132 | Neon-Mss2 3_Fwd BasI | GGACCGGGTCTCTAGCGACCGTAATATAAATCTTGTATT |
| 9133 | Mss2-Neon GGA-mNeon 5_fwd | GGACCGGGTCTCTTTGTAGGTGGTAGTGGTATGGTTTCTA |
| 9134 | Mss2-Neon mNeon 3_Rev | GGACCGGGTCTCTCGCTCTAGAACTAGTGGATCT |
| 9135 | Neon-Mss2 3_Rev GGA BasI | GGACCGGGTCTCTATGGTTAATTAAATTATTATATGTATGT  CTTTGTGGGTTCC |
| 2664 | Sac1 Dox F (SalI) | GGACCGGTCGACAAAGATGGTGTTAACCCATTCCACA |
| 2665 | Sac1 Dox R (BamHI) | GGACCGGGATCCTCAATTCTTTTTCTTATTATTAACTGTGAAATTTTCT |
| 2666 | Ume6 Dox F (Not1) | GGAGCGGCGGCCGCATGATTACCCATATGGTTACACCCG |
| 2667 | Ume6 Dox R (StuI) | GGAGCGAGGCCTTCAATCATTGGTTATATCATTACTTGATTT |
| 2668 | ORF19.1841 Dox F (Not1) | GGAGCGGCGGCCGCATGTCCGAAAACACATCACAAGAAT |
| 2669 | ORF19.1841 Dox R (StuI) | GGAGCGAGGCCTTCACTTAGTTAACTCCACAATAAATTGTGC |
| 2670 | Rim8 Dox F (Not1) | GGAGCGGCGGCCGCATGAGACGAGCAGTATCAAAAATACTACC |
| 2671 | Rim8 Dox R (AflII) | GGAGCGCTTAAGTTACGTCTCTGAATTCGAGTTATTATCC |
