# Supplementary figures and images for: Mss2 shapes the virulence of Candida albicans through reactive oxygen species (ROS) and calcium signaling, independent of direct transcriptional control
Source: Virulence. 2025 Nov 20;16(1):2590329. doi: 10.1080/21505594.2025.2590329 (PMC12645863; doi:10.1080/21505594.2025.2590329)

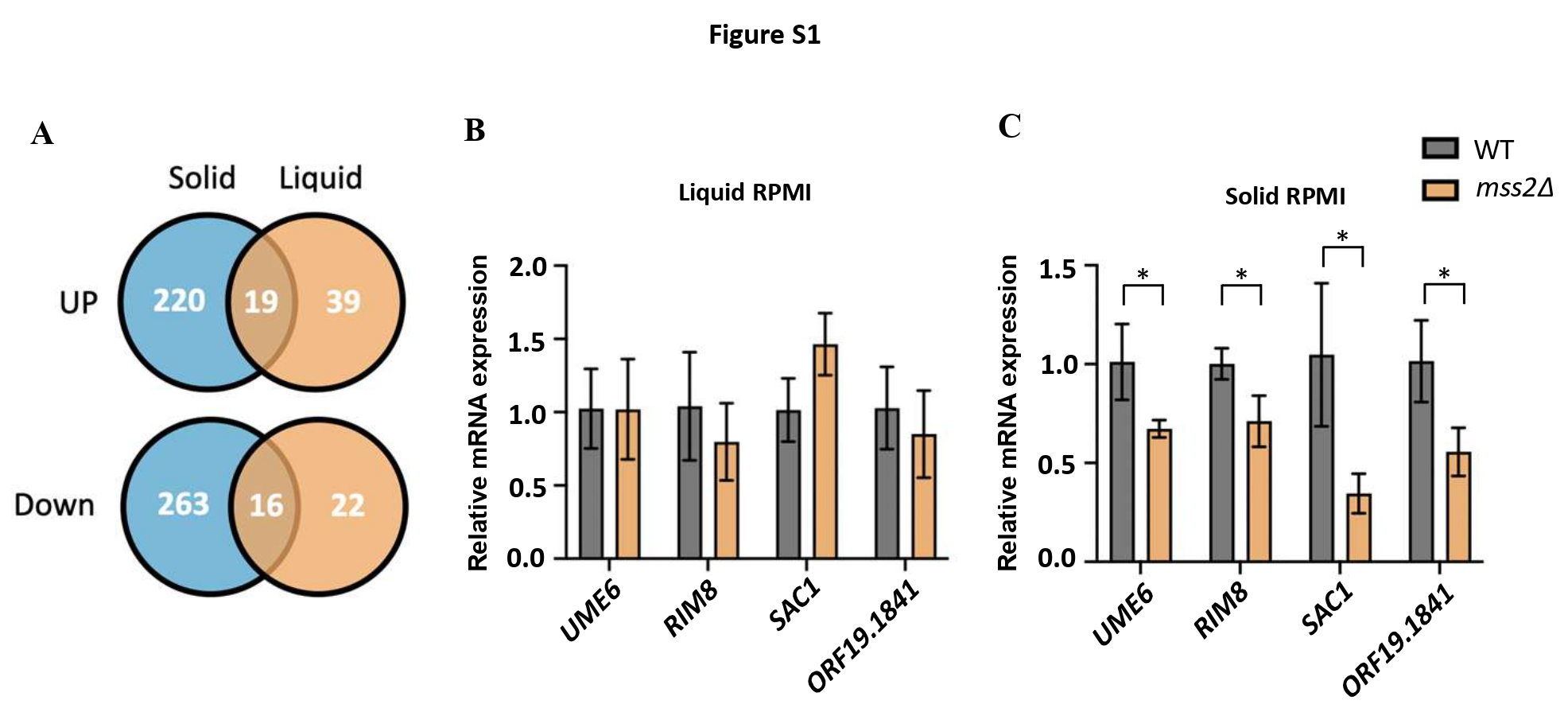

Supplement: Figure S1.tif [file KVIR_A_2590329_SM4204.tif]
